# Implementation of an Internet Weight Loss Program in a Worksite Setting

**DOI:** 10.1155/2016/9372515

**Published:** 2016-01-28

**Authors:** Kathryn M. Ross, Rena R. Wing

**Affiliations:** Department of Psychiatry and Human Behavior, Alpert Medical School of Brown University and The Miriam Hospital, Providence, RI 02906, USA

## Abstract

*Background.* Worksite wellness programs typically produce modest weight losses. We examined whether an efficacious Internet behavioral weight loss program could be successfully implemented in a worksite setting.* Methods.* Participants were 75 overweight or obese employees/dependents of a large healthcare system who were given access to a 12-week Internet-based, multicomponent behavioral weight loss program. Assessments occurred at baseline, Month 3 (end of intervention), and Month 6 (follow-up).* Results.* Retention was excellent (93% at Month 3 and 89% at Month 6). Intent-to-treat analyses demonstrated that participants lost an average (±SE) of −5.8 ± .60 kg from baseline to Month 3 and regained 1.1 ± .31 kg from Month 3 to Month 6; overall, weight loss from baseline to Month 6 was −4.7 ± .71 kg, *p* < .001. Men lost more weight than women, *p* = .022, and individuals who had a college degree or higher lost more weight than those with less education, *p* = .005. Adherence to viewing lessons (8 of 12) and self-monitoring (83% of days) was excellent and significantly associated with weight loss, *p*s < .05.* Conclusions.* An Internet-based behavioral weight management intervention can be successfully implemented in a worksite setting and can lead to clinically significant weight losses. Given the low costs of offering this program, it could easily be widely disseminated.

## 1. Introduction

With two-thirds of the United States population considered overweight or obese [1], there is a need for effective weight loss interventions that can be widely disseminated at minimal cost. Traditional behavioral weight loss programs, which typically include weekly or biweekly in-person or group-based meetings with a trained interventionist, are effective in helping obese and overweight individuals lose weight [2], but the reach of these programs is often limited due to high cost and time burden for both providers and participants. Consequently, efforts have been made to increase the dissemination potential of these interventions through alternate delivery modalities such as Internet-based programs. Early research on Internet-based weight management programs, however, demonstrated weight losses that were inconsistent and smaller than those typically observed in traditional face-to-face behavioral interventions [3]. Despite these early findings, more recent research has demonstrated that integrating additional treatment components (such as interactive multimedia lessons, small financial incentives, and automated tailored feedback based on participants' goal progress) demonstrates promise for improving the efficacy of Internet-based behavioral weight management programs [4–6]. Two recent studies investigating the use of a 12-week Internet-based behavioral weight loss program with small financial incentives [5] and secondarily in a primary care setting [7] demonstrated weight losses of 6.4% and 5.8% of baseline weight at immediate posttest (3 months), respectively, with maintenance of these weight losses at 6 months.

Given the efficacy of newer Internet-based weight management programs, an important next step is to identify avenues for dissemination. Corporate wellness programs offer an ideal platform for the dissemination of evidence-based weight management programs, as corporations have vested financial interest in improving employee health. Obesity costs employers up to $73.1 billion dollars per year in medical costs and absenteeism; per employee costs range from $322 to $6087 (from overweight to the highest category of obesity) in men, and from $797 to $6694 in women [8]. A 2012 survey found that almost 80% of companies employing over 1,000 people had corporate wellness programs [9], and this number is likely to increase with funding provisions in the Affordable Care Act [10].

Systematic reviews of worksite-based behavioral interventions suggest that these programs typically produce statistically significant but modest weight losses [11, 12]. In a recent review, Anderson and colleagues reported a pooled estimate for weight loss of −1.27 kg (range: +1.63 kg to −6.70 kg compared to control) at either 6- or 12-month follow-up compared to control across nine randomized controlled trials in worksite settings [11]. Moreover, Internet programs implemented in this setting have had limited efficacy, with 7 of 15 studies showing no significant changes in body weight [13]. Thus, it is important to determine whether a specific Internet program that has been shown to produce weight loss in other settings can be effectively implemented within a worksite or corporate wellness program.

For the current study, we used an Internet-based behavioral weight management that has been demonstrated to be efficacious within a community-level health program [4, 5] and in a primary care medical setting [7] (with mean weight losses of 6% over the 3-month program in these settings) and investigated its implementation within an existing corporate worksite wellness program. We report on the average weight losses achieved, the percent of participants who obtained a clinically significant weight loss (≥5% of baseline body weight), and whether the program was differentially effective for specific subgroups [14].

## 2. Methods

### 2.1. Participants

Participants were employees or dependents of employees who were enrolled in the worksite healthcare reward program of a large healthcare corporation in Providence, Rhode Island. Recruitment was limited to 100 eligible individuals. Potential participants (between the ages of 18 and 70 years and who had BMIs of at least 25 kg/m^2^) were contacted in one of two ways: (1) by the healthcare rewards program (separate from the research team) through targeted e-mails and texts and (2) through advertisements placed on the worksite intranet. The emails, texts, and advertisements instructed participants to provide their name and email on a hospital voicemail system, after which they were sent an email with a unique link to our study website. This link provided additional information about the study and allowed participants who were still interested to complete a prescreen questionnaire that assessed basic eligibility criteria (e.g., age, BMI, or self-report of medical conditions that would contraindicate weight loss). After completing this prescreen, potentially eligible individuals were invited to schedule an in-person orientation visit at the Weight Control and Diabetes Research Center (WCDRC) in Providence, Rhode Island. This visit provided potential participants with a thorough introduction to the study and research procedures, after which written informed consent was collected. After providing consent, participants had their height and weight measured and were asked to complete baseline assessment questionnaires.

Potential participants were excluded if their weight was >150 kg (a restriction of in-home body weight scales provided as part of the study), if they were unable to attend the assessment visits, if they reported that they were currently pregnant or planned to become pregnant in the next 12 months, if they were currently enrolled in another weight loss program or research study or had completed a study at our center within the past 2 years, or if they did not have access to a computer/Internet at home. Further, participants with medical conditions that would contraindicate weight loss behaviors (e.g., uncontrolled hypertension or diabetes, undergoing treatment for cancer, recent history of coronary heart disease, self-report of an eating disorder, inability to walk at least 2 blocks without stopping, or weight loss of ≥4.5 kg in the month prior to enrollment) or factors that would render the participant unlikely to complete the study (e.g., plans to relocate, substance abuse, terminal illness, severe psychiatric conditions, or dementia) were excluded. Approval for this study was obtained from the Miriam Hospital Institutional Review Board.

### 2.2. Procedure

All participants were provided with a 12-week, multicomponent Internet-based lifestyle weight management program that combined an initial hour-long, intensive in-person group visit with an Internet-based intervention, in-home body weight scale, paper food records, financial incentives for self-report of weekly data, and optional in-person counseling sessions if participants achieved only minimal weight loss at 4 weeks. This program has demonstrated efficacy for weight loss in community and primary care settings [5, 7].

The program started with an in-person group visit, at which participants learned how to use the study website and were given basic education regarding weight management and prescribed calorie, dietary fat, and physical activity goals. All participants were instructed to consume 1200–1800 kcal/day, depending on their initial body weight, and to reduce dietary fat intake to less than 30% of total daily calories. Participants were further instructed to gradually increase their engagement in moderate-intensity physical activity (primarily through brisk walking), eventually reaching a goal of 200 minutes per week. Finally, participants were taught how to self-monitor caloric intake, fat intake, and physical activity and how to enter this information into the study website. Participants were given an in-home body weight scale that transmitted their weight data to the WCDRC, a calorie reference book, and paper food records to use during the 12-week weight management program.

#### 2.2.1. Internet-Based Program

The Internet-based program was delivered weekly over a 12-week period. At the beginning of each week, participants were provided with a new, 15-minute multimedia lesson. These lessons presented standard behavioral weight loss strategies and were adapted from the Diabetes Prevention Program [15], Look AHEAD [16], and other behavioral weight loss programs. To increase participant engagement, the interactive lessons incorporated video, animation, audio, quizzes, and exercises for goal setting and problem-solving [17]. While only one new lesson was provided per week, participants could view previous weeks' lessons at any time.

A key component of the program was self-monitoring and participants were asked to record their daily weight, calorie and fat intake, and minutes of physical activity. Participants were asked to submit their self-monitoring data at least once a week and received tailored, automated feedback (generated using an algorithm) related to their goals for weekly and overall weight loss, caloric intake, and physical activity minutes. Reinforcement and support were provided for goals that were met, and encouragement, along with specific behavioral strategies to try, was provided for goals which were unmet. The website also provided a chart displaying a participant's weight change to date, healthy recipes, and additional weight control information that they could access if desired. The Internet program lasted for 3 months, after which participants no longer had access to the intervention website (i.e., the weight chart, intervention lessons, and other intervention materials). Between months 3 and 6 participants were encouraged to continue to self-monitor caloric intake, minutes of physical activity, and body weight but no feedback was provided.

As previous literature has demonstrated that initial weight loss predicts long-term outcome, participants who had lost only small amounts of weight (<2.0% of initial body weight) by the end of week 4 of the Internet-based intervention (*n* = 15) were contacted and given the opportunity to have a one-time, in-person counseling session that focused on problem-solving. Participants were given samples of portion-controlled frozen food entrees (e.g., Lean Cuisine or Healthy Choice meals) and a structured meal plan with similar meal replacements. A brief follow-up phone call was provided one week later; no additional contact was provided following this brief intervention.

#### 2.2.2. Incentives

As part of an existing corporate healthcare rewards program, all employees and dependents of the healthcare corporation were eligible to receive $250 for completing an annual physical with their physician and either (a) having a BMI ≤ 30 kg/m^2^ or (b) having lost either ≥5% of starting weight or at least 17 lbs, between the initial physician visit and October 31st of the calendar year. The current weight loss intervention was offered as one of the programs through which reward program participants could lose weight toward this goal.

As previous research on the impact of adding small financial rewards to an Internet-based weight management program demonstrated superior weight losses [5], and these rewards (an average of $3.50 per week per participant) were deemed acceptable to the healthcare reward program, the current study provided small financial incentives in return for the submission of weekly self-monitoring data and completion of a brief questionnaire. Incentives varied from week to week (range from $1 to $10) on a schedule unknown to participants, with a total possible incentive of $86 over the 6 months. Participants collected incentives in an online “bank,” which were redeemable at the assessment visits.

### 2.3. Measures

Assessments were completed at baseline, Month 3 (end of the 12-week Internet program), and Month 6 (after 3 months of follow-up with no further intervention). Weight was the primary outcome in the current study and was measured to the nearest 0.1 kg at each assessment, using a calibrated digital scale and with participants in light indoor clothing and no shoes. Height was measured at baseline using a wall-mounted stadiometer, with shoes removed, to the nearest 0.1 cm. Basic demographic data were collected at baseline using a self-report questionnaire. Adherence to the program goals was assessed using self-monitoring data submitted weekly via the study website. During weeks 1–12, participants were asked to log in once each week to submit their daily weight, caloric intake, fat intake, and minutes of physical activity. From weeks 13 to 24, participants were asked to log on once each week to self-report the number of days each week that they weighed themselves, the number of days that they tracked their caloric intake, and their total minutes of physical activity during the week. Finally, participant engagement with the Internet-based program was assessed through number of log-ins to the study website and number of video lessons watched.

### 2.4. Analyses

Independent samples *t*-tests and Chi-square tests (using Fisher's exact test when appropriate due to cell sizes) were used to assess differences between participants who did and who did not return for the Month 6 assessment. All participants who began the study were included in all analyses (an intent-to-treat approach). Weight change over time was assessed with paired samples *t*-tests. Multiple imputation (using the Markov Chain Monte Carlo method) was used to handle missing data. We further replicated our results using baseline observation carried forward (BOCF), wherein individuals who did not return for follow-up visits were assumed to have returned to their baseline weight. Given a similar pattern of results, the results from the analyses using multiple imputation for missing data are presented.

Descriptive statistics were used to investigate the attainment of clinically significant weight loss (≥5% of baseline weight) at Months 3 and 6 (participants who did not return for follow-up were assumed to have not met this threshold). Linear regression models were used to assess differences in percent weight change from baseline to Month 6 between different subgroups of participants, including those who were overweight compared to those who were obese at baseline, and other potential treatment modifiers (gender, age, race/ethnicity, marital status, and education). Website usage and adherence data were presented using descriptive analyses. Pearson correlations were used to investigate the associations between self-monitoring variables, and further between adherence to self-monitoring and percent change in weight from baseline to Month 3. All analyses were conducted using SAS version 9.4 for Windows [18].

## 3. Results

**Figure 1:**
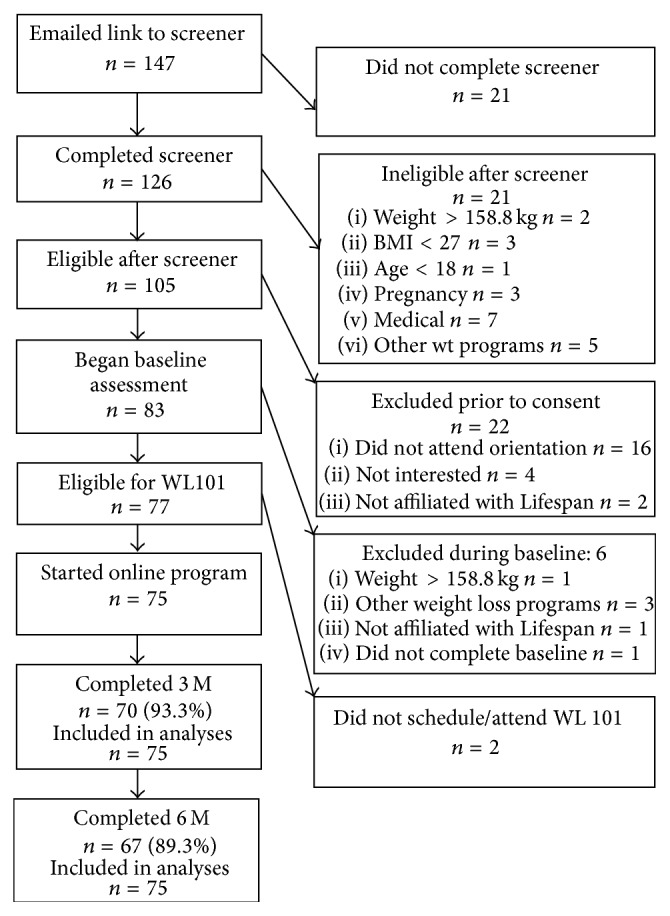
Participant flow through recruitment and intervention.

Of the 147 potential participants who provided their name and email address to the research team, 105 were scheduled for orientation/baseline assessment (after which scheduling was stopped due to a recruitment goal of *N* = 100) and 75 enrolled in the current study; see Figure 1 for participant flow through recruitment and assessment. Seventy of the 75 participants (93.3%) returned for the Month 3 assessment and 67 (89.3%) returned for the Month 6 assessment. Baseline and demographic data are presented in Table 1. There were no differences between participants who did and did not return for the Month 6 assessment in terms of age (*p* = .750), sex (*p* = .532), or race/ethnicity (*p* = .623). There was a significant difference in attrition by education, such that individuals who did not return for the Month 6 assessment were less likely to have a college or graduate degree (Fisher's exact *p* = .001).

### 3.1. Weight Change

On average, participants lost a mean (±SE) of −5.78 ± 0.60 kg (−6.37 ± 0.60% from baseline) during the course of the 12-week intervention, *t*(74) = −9.70, *p* < .0001. From Month 3 to Month 6, participants regained an average of 1.10 ± .31 kg (1.39 ± 0.40%), *t*(74) = 3.60, *p* = .009. Overall, from baseline to Month 6, participants lost a total of −4.68 ± 0.71 kg (−5.03 ± 0.76%), *t*(74) = −6.60, *p* < .0001. Mean changes with BOCF were almost identical. The 15 participants who were given additional support had a mean weight loss of −0.30 ± 1.19% at 4 weeks, which increased to −1.64 ± 1.50% at 6 weeks; however, weight loss for these participants at 6 months was 0.27 ± 1.86%, far below the −6.22 ± 0.80% experienced by the 60 participants who did not receive additional intervention.

We also examined the percent of participants who achieved a clinically significant weight loss of at least 5% of initial body weight [14]. At the end of the 12-week intervention, 60.0% of the sample (*n* = 45 of the 75) experienced a weight loss of ≥5%. Further, following a 3-month maintenance period wherein participants received no further contact or intervention, 53.3% of participants (*n* = 40 of the 75) maintained a weight loss of ≥5% at 6 months.

Percent weight loss from baseline to Month 6 did not differ between participants considered “overweight” (BMIs between 25.00 and 29.99 kg/m^2^) at baseline compared to those who were considered “obese” (BMIs above 30.00 kg/m^2^) at baseline, *p* = .666; participants who were overweight at baseline lost an average (±SE) of −4.67 ± 1.12% of their baseline weight, compared to a −5.33 ± 1.53% loss experienced by participants who were obese at baseline. Investigating other baseline factors, there was a significant association between sex and percent weight loss, such that men had a significantly greater percent weight loss than women from baseline to Month 6 (−7.69 ± 1.35% versus −3.85 ± 1.61%), *t*(74) = 2.39, *p* = .020. Further, there was a significant association between baseline education and percent weight loss from Months 0 to 6; participants who reported attaining a college degree or higher lost significantly more weight than those who reported less than a college degree (mean ± SE weight change = −6.41 ± 1.62% versus −1.71 ± 1.40%, resp.), *t*(74) = 2.90, *p* = .005. There was no difference in percent weight change from baseline to Month 6 by age, race/ethnicity, income, or marital status, all *p*s > .05.

### 3.2. Use of the Internet Program

Table 2 provides data on user engagement (measured via website log-ins and video lessons viewed per person) and adherence to self-monitoring of weight, caloric intake, and physical activity, and the association between these factors and percent weight change from baseline to Month 3. As shown, participants viewed on average 8 of the 12 lessons and self-reported their weight on the website on 83% of the days; adherence to both of these aspects of the program was associated with weight loss.

## 4. Discussion

The current study investigated the impact of a multicomponent, Internet-based behavioral weight management program on weight loss in a workplace setting. Participants in the current study lost an average of 6.4% of their baseline weight during the 12-week intervention, and maintained a loss of 5.0% of their baseline weight at a follow-up at 24 weeks from baseline (Month 6). Sixty percent of the participants were able to lose at least 5% of their body weight, and despite experiencing some regain in the 3 months following intervention, over half were able to maintain a clinically significant weight loss at a three-month follow-up (Month 6).

Many eHealth and Internet-based weight management programs have suffered from low program engagement and adherence [17]; however, website utilization in the current study was high. Participants watched on average 8 of the 12 lessons and submitted their weight on 83% of the days during the initial 3-month program. Both the number of video lessons viewed and adherence to self-monitoring (via self-monitoring of weight, caloric intake, and physical activity) were significantly associated with weight loss at the end of the intervention.

The clinically significant weight losses observed coupled with the high participant engagement in the current study support the use of the current intervention package in a workplace setting. Moreover, the results suggest that the program was as efficacious in the worksite setting as it was in primary care and community programs [4, 5, 7]. Based on a prior series of programmatic studies, the following key components of the current Internet program used in community settings have been identified. The video lessons were shown to have only a small impact on weight losses when presented as a single component, but lessons combined with the provision of weekly automated feedback to participants was demonstrated to significantly improve weight loss outcome [6]. Adding small weekly incentives for self-monitoring adherence further improved these results [5]. Thus, these aspects of the program may also have been related to its success in the worksite setting.

The current study demonstrates that a low-intensity, multicomponent Internet-based approach may be beneficial in a workplace setting. Further, this intervention was particularly effective in men. Although gender differences are often seen in mean weight losses, adjusting for baseline weight typically removes this difference. In the present study, men had a 7.7% reduction in body weight compared to 3.9% in women, suggesting that this type of program may be particularly effective for men. As behavioral weight management studies typically have a higher enrollment of women compared to men, future studies should seek to increase the recruitment of men since this program appears particularly effective for these individuals.

Strengths of the current study include minimal attrition, objective measures of body weights at assessments, and the use of intent-to-treat analysis to account for missing data. Whereas many investigations into the efficacy of worksite weight loss programs have suffered from high attrition and failure to adjust for this attrition in subsequent analyses (e.g., using completers-only analyses) [12], the current study demonstrated good retention and conducted a conservative, intent-to-treat analyses; both multiple imputation and baseline observation carried forward approaches were used to manage missing data. Limitations to the current study include the lack of a no-treatment control group and the inability to assess relative impact of program components. The current study was procedurally limited in that the healthcare system that funded the intervention requested a weight management program that would be open to any eligible employee and would not involve the possibility of randomization to a control group or to programs with different components (which could then differ in their efficacy). Because there was no control group included in the current study, however, we cannot rule out other factors beyond intervention participation that may affect weight change over time. Additionally, participants self-selected to enroll in this trial, which may lead to a sample of highly motivated individuals; without including a no or minimal treatment control condition, we cannot control for the impact of self-selection on weight loss.

Finally, the current study did not include a maintenance component following the end of initial weight loss intervention (Month 3). Since research has demonstrated that providing extended-care interventions following the end of weight loss treatment programs improves long-term weight loss outcomes [19], a next step would be to develop and test an Internet-based maintenance component. Previous studies have demonstrated that an Internet-based maintenance component led to similar maintenance of weight loss (following an interactive-TV based weight loss intervention) when compared to a group that continued to meet face-to face [20]; it is unknown whether an Internet-based maintenance component would have similar impact following an Internet-based weight loss program.

## 5. Conclusion

The current study demonstrated that a 12-week, Internet-based weight loss intervention can be successfully implemented in a workplace setting and produce clinically significant weight losses. Participant engagement with the intervention website was high, as was adherence to self-monitoring of weight, caloric intake, and physical activity. Future studies should examine ways to improve the long-term maintenance of weight loss following the use of an Internet-based behavioral intervention in a workplace setting and determine the effect of the intervention on outcomes of particular interest to worksites, including medical expenditures and workplace productivity.

## Figures and Tables

**Table 1:** Baseline and demographic characteristics.

Variable	Total sample	
	*N* = 75	
	Mean	SD

Age, years	50.76	10.38
Weight, kg	86.42	1.94
BMI, kg/m^2^	31.19	4.41

	*n*	*%*

Gender		
Female	52	69.3
Male	23	30.7
Ethnicity (%)		
African American	4	5.3%
Asian	1	1.3%
Caucasian	63	84.0%
Hispanic	2	2.7%
Other/multiple	5	6.7%
Marital status		
Single	5	6.7%
Married or living with a partner	61	81.3%
Separated/divorced	9	12.0%
Household income, dollars		
25,000–50,000	7	9.3%
50,001–75,000	16	21.3%
75,001–100,000	18	24.0%
100,001–125,000	10	13.3%
125,001+	22	29.3%
Not reported	2	2.7%
Education		
High school or less	6	8.0%
Vocational training	2	2.7%
Some college	14	18.7%
College or university degree	34	45.3%
Graduate degree	19	25.3%

**Table 2:** Website engagement and adherence to self-monitoring during the intervention, and the association between these factors and weight loss at Month 3.

	Frequency per participant		Correlation with percent weight change	
	*n*	SD	*r*	*p*

Website log-ins	39.65	27.94	−.098	.438
Video lessons viewed^*∗*^	8.05	3.44	−.319	.009

	%	SD		

Self-reporting body weight	83.24	23.8	−.362	.034
Self-reporting caloric intake	82.44	24.04	−.374	.030
Self-reporting physical activity	80.37	35.85	−.409	.014

^*∗*^Of 12 video lessons.
